# The effect of empagliflozin on arterial stiffness and heart rate variability in subjects with uncomplicated type 1 diabetes mellitus

**DOI:** 10.1186/1475-2840-13-28

**Published:** 2014-01-29

**Authors:** David ZI Cherney, Bruce A Perkins, Nima Soleymanlou, Ronnie Har, Nora Fagan, Odd Erik Johansen, Hans-Juergen Woerle, Maximilian von Eynatten, Uli C Broedl

**Affiliations:** 1 Division of Nephrology, University Health Network, University of Toronto, Toronto General Hospital, 585 University Ave, Toronto 8N-845, M5G 2N2, Ontario, Canada; 2 Division of Endocrinology, University Health Network, University of Toronto, Toronto, Canada; 3 Boehringer Ingelheim Canada Ltd./Ltée, Burlington, Canada; 4 Boehringer Ingelheim Pharmaceuticals, Inc., Ridgefield, CT, USA; 5 Boehringer Ingelheim Pharma GmbH & Co.KG, Ingelheim, Germany

**Keywords:** Diabetes mellitus, Systemic blood pressure, SGLT2 inhibition, Empagliflozin, Hyperglycaemia, Arterial stiffness, Heart rate variability

## Abstract

**Background:**

Individuals with type 1 diabetes mellitus are at high risk for the development of hypertension, contributing to cardiovascular complications. Hyperglycaemia-mediated neurohormonal activation increases arterial stiffness, and is an important contributing factor for hypertension. Since the sodium glucose cotransport-2 (SGLT2) inhibitor empagliflozin lowers blood pressure and HbA1c in type 1 diabetes mellitus, we hypothesized that this agent would also reduce arterial stiffness and markers of sympathetic nervous system activity.

**Methods:**

Blood pressure, arterial stiffness, heart rate variability (HRV) and circulating adrenergic mediators were measured during clamped euglycaemia (blood glucose 4–6 mmol/L) and hyperglycaemia (blood glucose 9–11 mmol/L) in 40 normotensive type 1 diabetes mellitus patients. Studies were repeated after 8 weeks of empagliflozin (25 mg once daily).

**Results:**

In response to empagliflozin during clamped euglycaemia, systolic blood pressure (111 ± 9 to 109 ± 9 mmHg, p = 0.02) and augmentation indices at the radial (-52% ± 16 to -57% ± 17, p = 0.0001), carotid (+1.3 ± 1 7.0 to -5.7 ± 17.0%, p < 0.0001) and aortic positions (+0.1 ± 13.4 to -6.2 ± 14.3%, p < 0.0001) declined. Similar effects on arterial stiffness were observed during clamped hyperglycaemia without changing blood pressure under this condition. Carotid-radial pulse wave velocity decreased significantly under both glycemic conditions (p ≤ 0.0001), while declines in carotid-femoral pulse wave velocity were only significant during clamped hyperglycaemia (5.7 ± 1.1 to 5.2 ± 0.9 m/s, p = 0.0017). HRV, plasma noradrenalin and adrenaline remained unchanged under both clamped euglycemic and hyperglycemic conditions.

**Conclusions:**

Empagliflozin is associated with a decline in arterial stiffness in young type 1 diabetes mellitus subjects. The underlying mechanisms may relate to pleiotropic actions of SGLT2 inhibition, including glucose lowering, antihypertensive and weight reduction effects.

**Trial registration:**

Clinical trial registration: NCT01392560

## Background

In type 2 diabetes mellitus (T2D), approximately 40% of patients are hypertensive at the time of diagnosis, and in type 1 DM (T1D), the prevalence of hypertension increases with longer duration of disease [1]. Hyperglycaemia plays a critical role in the pathogenesis of diabetic complications and in the development of hypertension in patients with DM. This is explained in part through effects on activation of the renin angiotensin aldosterone system (RAAS) and sympathetic nervous system (SNS) as well as suppression of nitric oxide [2], leading to macrovascular dysfunction including increased arterial stiffness [3]. Together with changes in arterial structure secondary to long-standing DM, hyperglycaemia-mediated neurohormonal activation increases vascular tone and arterial stiffness, thereby raising the risk of hypertension [4,5]. Unfortunately, blockade of RAAS pathways provides incomplete protection against the development of diabetic complications [6] and dual RAAS blockade strategies may increase the risk of serious adverse effects such as acute kidney injury and hyperkalemia [7,8]. The development of safe, new agents that augment vascular protection in patients with DM is therefore of the utmost importance.

Aside from effects of RAAS and SNS activation on vascular function, arterial stiffness increases under the influence of ambient hyperglycaemia and can be improved through tight glycemic control [9-11]. Intensification of glycemic control in both T1D and T2D reduces arterial stiffness, which may contribute to improved blood pressure control and a decreased risk of cardiovascular complications [12-14]. Unfortunately, intensive glucose lowering strategies also increase the risk of severe hypoglycemic events [15-17] and promote weight gain and sodium retention resulting in higher blood pressure [16-18], thereby mitigating the benefit that can be achieved through tight glycemic control [19].

**Figure 1:**
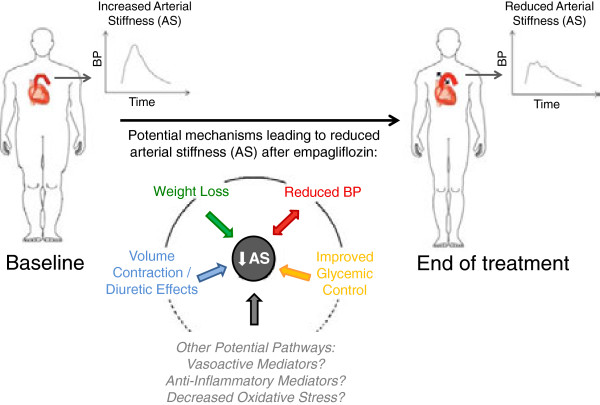
Physiological mechanisms implicated in arterial stiffness lowering effects with sodium glucose cotransport-2 inhibition.

Alternatively, glucose lowering may be achieved with novel sodium glucose co-transport 2 (SGLT2) inhibitors in patients with T2D [20,21]. Since these agents lower blood glucose through insulin-independent increases in urinary glucose excretion, SGLT2 inhibition has also been used in T1D patients, and improves glycemic control [22,23]. In addition to having a low risk of hypoglycaemia, this class of oral hypoglycemic agents induces clinically relevant and sustained weight loss and declines in blood pressure in patients with T1D [22,24] and T2D [20,25]. The blood pressure lowering effect of SGLT2 inhibition may be related to several mechanisms, including diuretic effects, changes in neurohormonal activation, improved glycemic control and decreases in body weight (Figure 1). Despite these pleiotropic effects, mechanistic human data related to antihypertensive effects of these agents remain limited. A thorough understanding of the blood pressure lowering effects of SGLT2 inhibition is important, since long-term cardiovascular outcome trials are underway, and human mechanistic data may help to interpret the results of these clinical trials.

In a trial designed with the primary objective of investigating renal hemodynamic effects of SGLT2 inhibition, we examined systemic hemodynamic effects of empagliflozin (Boehringer Ingelheim, Ingelheim, Germany), a new and highly selective SGLT2 inhibitor. We hypothesized that empagliflozin would decrease arterial stiffness, including augmentation index and pulse wave velocity, leading to declines in systemic blood pressure. In addition, due to the stimulatory effect of hyperglycaemia on the SNS [26], we hypothesized that empagliflozin would reduce neurohormonal activation, leading to improved heart rate variability (HRV) and a decline in plasma adrenaline and noradrenalin.

## Methods

### Subjects

In this 8-week, open-label, prospective clinical trial, 42 participants with T1D were treated with empagliflozin 25 mg once daily (NCT01392560). Inclusion and exclusion criteria at screening have been described elsewhere [24] (http://www.clinicaltrials.gov/ct2/show/NCT01392560). In brief, men and women ≥18 years with T1D ≥ 12 months with estimated GFR ≥60 ml/min/1.73 m^2^ and no hypertension or macroalbuminuria were included. Patients taking medication known to interfere with RAAS activity and/or renal function based on investigator judgment were excluded [24]. Of note, none of the patients were taking medications that can affect arterial stiffness or blood pressure, such as renin angiotensin aldosterone system blockers, beta blockers, calcium channel blockers, diuretics or other antihypertensive agents. Two cases of diabetic ketoacidosis leading to discontinuation occurred during the study: one in the context of insulin pump failure and the other in the setting of acute gastroenteritis. These patients were withdrawn within the first 3 days of drug exposure and were included only in the safety analyses. Detailed data regarding other adverse effects of empagliflozin in this cohort have been reported elsewhere [24]. 40 subjects completed the study (Table 1) and the primary endpoint of change in renal function in response to empagliflozin has been reported elsewhere [24]. In this manuscript we report the pre-specified exploratory vascular endpoint results. The Research Ethics Board at the University Health Network (Toronto, Canada) approved the protocol and all subjects gave informed consent prior to start of study procedures. The study was conducted according to the International Conference on Harmonization on Good Clinical Practice.

**Table 1:** Baseline Clinical Characteristics of the 40 Patients with Type 1 Diabetes

Male sex (%)	20 (50%)
Age (yr – mean ± SD)	24.3 ± 5.1
Diabetes Duration (yr)	
>1-5 years – n (%)	4 (10)
>5 years – n (%)	36 (90)
Current Smoking - n (%)	4 (10)
Presence of Non-Proliferative Retinopathy - n (%)	0
Body mass index (kg/m^2^)	24.5 ± 3.2
HbA1C (%)	8.0 ± 0.9
Urine albumin/creatinine ratio (mg/mmol)	1.2 ± 0.9

Urine albumin/creatinine ratio reported is the average of the baseline values that were collected during clamped euglycemia and hyperglycemia. Urinary albumin concentration was determined by immunoturbidimetry and HbA1C was measured by high-performance liquid chromatography [27].

### Experimental design

Subjects adhered to a sodium-replete and moderate protein diet during the 7-day period before each experiment, as described previously [27]. After admission to the Renal Physiology Laboratory, euglycemic (4–6 mmol/L) conditions on the first day, followed by hyperglycemic (9–11 mmol/L) conditions on the second day, were maintained by a modified glucose clamp technique. Studies were performed on two consecutive days at baseline after maintenance of the clamp within the desired range for approximately 5 hours preceding and during all investigations, as described previously [24].

Following the glucose clamp, right radial artery and carotid waveforms were recorded with a high-fidelity micromanometer and using the validated transfer function, corresponding central aortic pressure waveform data were generated (SPC-301, Millar Instruments SphygmoCor, AtCor Medical Systems Inc., Sydney, Australia). Augmentation index, an estimate of systemic arterial stiffness was calculated as the difference between the second systolic peak and inflection point, expressed as a percentage of the central pulse pressure corrected to an average heart rate of 75 beats per minute. The aortic pulse wave velocity (PWV) was measured using the same device by sequentially recording ECG-gated right carotid and radial artery waveforms. Two vascular measurements were obtained for each parameter and the average value reported. The use of the SphygmoCor device to assess arterial stiffness parameters has been previously published by our group [28]. Automated blood pressure measurements were obtained using a DINAMAP machine (Critikon, Tampa, Florida) and the average of two values obtained immediately prior to the arterial stiffness assessments are reported.

After completion of arterial stiffness testing, HRV testing was performed using AtCor software (Atcor Medical Systems Inc., Sydney, Australia). In brief, two 10-minute segments were recorded. Vagal tone (Root Mean Square Successive Difference - RMSSD) and sympathetic activity (Standard Deviation of Normal-to-Normal interval - SDNN) measures were obtained at each of the two periods and the results were then averaged. Plasma noradrenalin and adrenaline concentrations were obtained on each of the study days according to standard, previously described methods [29].

### Statistical analysis

The primary endpoint of this study was change in GFR after treatment with empagliflozin for 8 weeks [24]. Sample size calculations were based on anticipated changes in GFR [24]. Accordingly, results reported in this manuscript were pre-specified, exploratory endpoints. Paired t-tests were performed to evaluate differences in vascular measurements and neurohormonal outcomes before and after treatment with empagliflozin. A repeated measures model was used to evaluate differences in response to empagliflozin between euglycemic and hyperglycemic conditions. All statistical analyses were performed using the statistical package SAS (Version 9.2).

## Results

### Baseline demographic parameters

The baseline characteristics of this T1D study cohort are described in Table 1. This cohort comprised a mainly young group of participants with uncomplicated T1D that has been described in more detail elsewhere [22,24]. All except for 4 patients (ages 31, 33, 34 and 44 years old) were between the ages of 18–30.

### Effects of empagliflozin on blood pressure, arterial stiffness and heart rate variability during clamped euglycaemia

**Figure 2:**
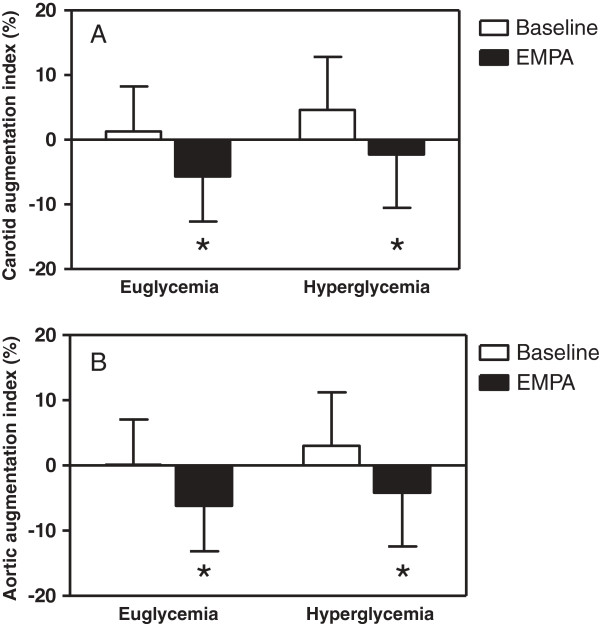
**The effect of empagliflozin on carotid (A) and aortic (B) augmentation indices during clamped euglycaemia and hyperglycaemia in patients with type 1 diabetes.** *p < 0.0001 compared to baseline parameter under the same glycemic condition.

During clamped euglycaemia, empagliflozin significantly reduced systolic blood pressure, radial augmentation index, carotid augmentation index and aortic augmentation index (Table 2, Figure 2). Carotid-radial pulse wave velocity decreased significantly (p = 0.0001), while similar trends for carotid-femoral pulse wave velocity were not significant. Effects on vagal tone (RMSSD) and SNS activity (SDNN, plasma noradrenalin and adrenaline) were not significant.

**Table 2:** Hemodynamic responses to empagliflozin in patients with type 1 diabetes during clamped euglycemia and hyperglycemia (mean ± SD)

	**Euglycemia**			**Hyperglycemia**		
	**Baseline**	**Empagliflozin**	**p-value**	**Baseline**	**Empagliflozin**	**p-value**
*Blood pressure*						
Systolic blood pressure (mmHg)	111.2 ± 8.9	108.5 ± 8.7	0.02	112.1 ± 9.8	110.6 ± 9.8	0.2797
Diastolic blood pressure (mmHg)	63.6 ± 8.5	63.1 ± 8.1	0.6191	65.2 ± 8.3	63.8 ± 7.3	0.2497
Pulse (beats per minute)	74.2 ± 13.1	71.8 ± 13.8	0.1885	72.0 ± 11.0	70.8 ± 12.8	0.4919
*Vascular parameters*						
Radial augmentation index (%)	-52.0 ± 16.1	-57.0 ± 16.7	0.0001	-47.9 ± 17.3	-52.1 ± 17.6	0.0190
Carotid radial pulse wave velocity (m/s)	7.3 ± 1.1	6.7 ± 0.9	0.0001	7.9 ± 1.1	6.9 ± 0.9	<0.0001
Carotid femoral pulse wave velocity (m/s)	5.5 ± 0.9	5.3 ± 1.0	0.1366	5.7 ± 1.1	5.2 ± 0.9	0.0017
*Heart rate variability*						
RMSSD (milliseconds)	46.3 ± 22.9	53.6 ± 32.1	0.1050	58.0 ± 35.1	63.3 ± 37.7	0.3004
SDNN (milliseconds)	69.3 ± 24.0	73.8 ± 33.2	0.3278	77.7 ± 32.8	83.4 ± 32.2	0.2837
*Biochemistry*						
Plasma adrenaline (nmol/L)	0.13 ± 0.07	0.12 ± 0.06	0.2946	0.13 ± 0.09	0.12 ± 0.06	0.1097
Plasma nordrenaline (nmol/L)	0.75 ± 0.35	0.76 ± 0.36	0.8985	0.77 ± 0.95	0.70 ± 0.35	0.6230

### Effects of empagliflozin on blood pressure, arterial stiffness and heart rate variability during clamped hyperglycaemia

During clamped hyperglycemic conditions, treatment with empagliflozin did not reduce blood pressure significantly, despite significant effects on radial augmentation index, carotid augmentation index and aortic augmentation index (Table 2, Figure 2). Empagliflozin decreased both carotid-radial and carotid-femoral pulse wave velocity under clamped hyperglycemic conditions (Table 2). Similar to effects during clamped euglycemic conditions, effects on SNS function including RMSSD, SDNN, plasma noradrenalin and adrenaline, were not significant (Table 2, Figure 2). In the repeated measures model used to evaluate differences in response to empagliflozin between glycemic conditions, there were no significant differences in any of the hemodynamic parameters (p > 0.05, data not shown).

## Discussion

Despite optimal medical therapy including RAAS inhibitors, many patients continue to develop hypertension, chronic kidney disease and cardiovascular diseases [6]. Moreover, in T1D, primary prevention strategies with RAAS blockers are ineffective [7]. Intensive glucose lowering therapies exert long lasting renal and cardiovascular protective effects, highlighting the need for improved glucose control in patients with T1D and T2D [30-32]. Due to their unique insulin-independent mechanism of action, SGLT2 inhibitors lower blood pressure and improve glycemic control, while at the same time avoiding potential risks of increased insulin doses such as hypoglycaemia, hypertension and weight gain [33]. These agents are also generally safe and well tolerated [22,34-39]. Our goal was to further clarify the mechanistic basis for the blood pressure lowering effect of empagliflozin, including effects on arterial stiffness and neurohormonal activation measured by heart rate variability, since both of these parameters are associated with systemic blood pressure and correlate with long-term renal and cardiovascular outcomes [5,40-42]. Our major novel finding was that empagliflozin reduced measures of arterial stiffness under both clamped euglycemic and hyperglycemic conditions. We also observed that after treatment with empagliflozin for 8 weeks, systolic blood pressure decreased significantly in normotensive patients with T1D.

### The effect of oral hypoglycemic agents on arterial stiffness

Previous studies in T2D have suggested that oral hypoglycemic agents such as metformin reduce blood pressure, primarily through improving glycemic control [12-14,43]. In contrast, other oral hypoglycemic agents such as sulfonylureas that increase the risk of severe hypoglycemic events and lead to body weight gain may promote the development of hypertension [44]. Perhaps as a result of increasing weight, these traditional oral hypoglycemic agents do not improve arterial stiffness parameters and a significant proportion of patients develop hypertension with increasing diabetes duration [45,46]. Since existing oral agents are not approved nor appropriate in T1D, the options for improving glycemic control in patients with T1D are limited to intensifying insulin regimens, which can have the same adverse effects on weight, hypoglycemic risk and blood pressure as in T2D patients [30-32]. This study shows for the first time that an oral hypoglycemic agent that improves glycemic control over an 8-week period in patients with T1D can also reduce arterial stiffness, which may in part be responsible for the antihypertensive effects associated with SGLT2 inhibition.

### Regulators of arterial stiffness

Arterial stiffness is influenced by a variety of factors, including neurohormonal mediators and changes in arterial structure [4]. We have previously demonstrated in this cohort that empagliflozin induces a modest increase in plasma RAAS mediators, including aldosterone and angiotensin II, which is likely a compensatory response to effective circulating volume contraction, similar to expected effects of a thiazide diuretic [24,47]. The mild increase in plasma RAAS levels with empagliflozin was insufficient to counteract the effect of effective circulating volume contraction, resulting in decreases in blood pressure and arterial stiffness. A similar observation was made for vasodilatory NO, for which we observed a decline in plasma levels in our previous work, which was also unlikely to have accounted for decreases in blood pressure or arterial stiffness in this cohort [24]. We therefore concluded that the beneficial hemodynamic changes observed with empagliflozin were unlikely to be influenced by concomitant changes in systemic RAAS and NO activity. Consequently we hypothesized that improved arterial compliance after empagliflozin could be related to changes in autonomic nervous system function, reflected by increased HRV and lower circulating levels of adrenaline and noradrenalin. However, empagliflozin did not affect HRV or plasma adrenergic system markers, suggesting that the mechanisms responsible for blood pressure and arterial stiffness lowering are independent of effects on autonomic nervous system activity. Nevertheless, our results do not exclude the possibility that other neurohormonal factors related to hyperglycaemia may have contributed to changes in blood pressure and arterial stiffness, such as decreased reactive oxygen species generation [48].

While neither RAAS, NO nor SNS activity can explain our results, we postulate that changes in arterial stiffness with empagliflozin are due to several other factors (Figure 1). First, weight loss independently decreases blood pressure and arterial stiffness [49]. Empagliflozin was associated with a significant reduction in weight of 2.7 kg in our cohort and this may have contributed to benefits on blood pressure and arterial stiffness, as previously reported [22,24]. This weight loss was likely in part due to loss of fat, since waist circumference declined. Second, decreases in daily insulin doses have been correlated with improved arterial compliance in T2D, and a similar interaction may have occurred in our T1D cohort since total daily insulin doses decreased significantly by the end of treatment [22,50]. Third, other diuretic agents such as thiazides lower arterial stiffness and these effects are likely in part due to direct effects on vascular smooth muscle relaxation after induction of a negative sodium balance [51,52]. Similar diuretic effects with SGLT2 inhibition may have contributed to the decline in arterial stiffness in our cohort. Next, empagliflozin-induced weight loss and improved glycemic control may exert anti-inflammatory changes, which favour improvements in blood pressure and arterial stiffness [49]. For example, in experimental models of diabetes, SGLT2 inhibition reduces oxidative stress and suppresses markers of inflammation and fibrosis, including nuclear factor κβ and collagen IV expression [53,54]. Due to the strong relationship between inflammation, cardiovascular complications and renal disease, future studies should clarify the ability of SGLT2 inhibitors to suppress inflammation in humans [55].

### Arterial stiffness and long term clinical outcomes

Due to their insulin-independent mechanism of action and as shown in animal models [56], SGLT2 inhibitors have the potential to be used in T1D [22] in addition to T2D [33]. Furthermore, SGLT2 inhibition may avoid some of the most common treatment-related side effects in T2D that have limited the benefits of traditional oral hypoglycemic agents, including significant weight gain, development and worsening of hypertension and severe hypoglycemic events [33,39,57]. Our observations are, to our knowledge, the first to suggest possible functional benefits of an oral hypoglycemic agent on large vessel function in T1D patients. The improvement in arterial compliance is important because previous studies have demonstrated strong, independent associations between macrovascular complications and arterial stiffness in T2D [58,59]. Increased arterial stiffness is also independently associated with clinically relevant outcomes in T1D patients, including cardiovascular, renal, retinal and autonomic complications [42]. Importantly, the beneficial effects of conventional cardiovascular protective agents such as ACE inhibitors have been in part attributed to improvements in arterial compliance [60]. Ultimately, large ongoing clinical outcomes trials in T2D will determine if changes in arterial stiffness or blood pressure with SGLT2 inhibition translate into long-term renal or cardiovascular protection.

This trial has some limitations. First, the duration of therapy was limited to 8 weeks. Therefore, conclusions regarding longer term clinical benefits cannot be made from this study. Second, further work is needed to explore the interdependence among beneficial effects on arterial stiffness, including weight loss, glycemic control and blood pressure seen with empagliflozin. In particular, we could not determine if changes in blood pressure were based on declines in arterial stiffness, or whether reduced arterial stiffness was responsible for the fall in systolic blood pressure. Nevertheless, consistent changes in these vascular parameters are encouraging and support the rationale for ongoing long-term cardiovascular outcome trials. Third, these results in young patients with uncomplicated T1D cannot be generalized to older T1D patients, T1D patients with hypertension or to those with T2D. Given the important relationship between age and arterial stiffness, future work should determine if SGLT2 inhibition exerts similar effects in older individuals. Finally, measures of arterial stiffness have a high variability. As a consequence, our results should be viewed as exploratory and should be confirmed in future studies.

## Conclusions

In summary, empagliflozin reduces arterial stiffness in patients with T1D. Longer-term studies are warranted to assess the safety and clinical effects of these agents in T1D. Moreover, since arterial stiffness is a surrogate marker for renal and cardiovascular clinical outcomes, future trials should assess the effect of empagliflozin on cardiorenal protection in patients with diabetes.

## Competing interests

The results presented in this paper have not been published previously in whole or in part. Some of the results presented in this paper were presented at the American Society of Nephrology Kidney Week in November 2013. D.Z.I.C. has received speaker honoraria from Boehringer Ingelheim and B.A.P received operational funding with D.Z.I.C. for this work. N.S., N.F., H.J.W. O.E.J., U.C.B., M.v E. are employees of Boehringer Ingelheim.

## Authors’ contributions

DZIC, BAP, RH, researched data, wrote the manuscript. NF, HJW, NS, OEJ, UCB, MvE contributed to discussion, reviewed/edited manuscript. All authors have approved the final version of this manuscript.

## Authors’ information

David ZI Cherney, Bruce A Perkins and Nima Soleymanlou are co-primary first authors and Maximilian von Eynatten and Uli C Broedl contributed equally as co-senior authors.
